# Dust Events and Indoor Air Quality in Residential Homes in Kuwait

**DOI:** 10.3390/ijerph17072433

**Published:** 2020-04-03

**Authors:** Yufei Yuan, Barrak Alahmad, Choong-Min Kang, Fhaid Al-Marri, Venkateswarlu Kommula, Walid Bouhamra, Petros Koutrakis

**Affiliations:** 1 Environmental Health Department, Harvard T.H. Chan School of Public Health, Harvard University, Boston, MA 02215, USA; b.alahmad@g.harvard.edu (B.A.); cmkang@hsph.harvard.edu (C.-M.K.); petros@hsph.harvard.edu (P.K.); 2 Environmental and Occupational Health Department, Faculty of Public Health, Kuwait University, 12037 Kuwait City, Kuwait; 3 Environmental Lab, Hawalli, Al-Rehab Complex, 36141 Kuwait City, Kuwait; kuwait@environmentallab.net (F.A.-M.); venkatk@environmentallab.net (V.K.); 4 President, Gulf University for Science and Technology (GUST), 32093 Kuwait City, Kuwait; bouhamraw@yahoo.com

**Keywords:** indoor air quality, Kuwait, particle penetration, dust storms, exposure assessment, indoor to outdoor ratio

## Abstract

Kuwait is a developed Middle Eastern country that is impacted by frequent dust storms from regional and/or remote deserts. The effectiveness of keeping homes tightly closed during these events to reduce dust exposures was assessed using indoor and outdoor particle samples at 10 residences within the metropolitan Kuwait City area. Specifically, this study compared indoor and outdoor levels of black carbon and 19 trace elements (Na, Mg, Al, Si, S, Cl, K, Ca, Ti, V, Cr, Mn, Fe, Ni, Cu, Zn, Br, Sr, and Zr) during dust and non-dust events and found that particle penetration efficiencies were lower during dust storm events (less than 20–30%) than during non-dust storm events (40–60%). Coarse particles had lower penetration efficiency compared to fine particles, which is due to differences in infiltration rates and settling velocities between these two size fractions. Our findings suggest that increasing home insulation could be an effective strategy to reduce indoor exposure to crustal particles from dust storm events in residential houses of Kuwait City.

## 1. Introduction

Over the past few decades, a large number of air pollution health effects studies have demonstrated the adverse effects of ambient (outdoor) particulate matter (PM) [1,2,3,4,5]. These studies have shown that PM exposures can impair human respiratory and cardiovascular health [6,7,8,9]. Therefore, assessing PM exposures is critical in developing cost-effective environmental policies to control air pollution emissions and to improve public health. 

Kuwait is a Middle Eastern developed country located at the northeast corner of the Arabian Peninsula and surrounded by the Persian Gulf. Because of its geographical location and climate change, it has been affected by droughts and frequent dust storms [10,11,12]. In recent years, regional military conflicts have contributed to more desertification in the area [13]. Several studies have examined the health effects of exposure to dust storms and have associated them with mortality and other outcomes [14,15,16,17]. To date, it is not well known which components of dust storms are responsible for the observed health effects. Possibly, dust storms serve as vectors of toxic elements and microorganisms. The effects of dust storms are exacerbated by the emissions from traffic as well as local and regional industrial sources, such as petrochemical industry, power plants, and cement industry, among others [17,18,19].

Unfortunately, natural sand dust cannot be regulated or prevented. Many dust storms originate from distant sources outside the country. Developing policies to protect the public from such natural pollutant sources can be challenging. It is common practice for residents of Kuwait City to keep their homes tightly closed during dust storm events to minimize dust entering indoors. Such practices include keeping windows and doors closed, taping the casings of windows, and placing wet towels under doors. Population exposure to the crustal particulate matter could be reduced by urging the public and sensitive subpopulations to stay indoors when dust storms are forecasted. However, there is very little information about the effectiveness of these practices. Previous indoor air quality studies in Kuwait have investigated school classrooms and homes [20,21,22,23,24]. Other studies in the region examined indoor and outdoor PM concentrations during dust storm events [25,26]. The harsh climate conditions in the Gulf led to the construction of tightly sealed, air-conditioned buildings [26]. However, very little is known about the impact of dust storms on indoor levels of fine (PM_2.5_) and coarse particles (PM_2.5–10_) in Kuwait. We employed custom-designed samplers to collect simultaneous indoor and outdoor samples of PM_2.5_ and PM_10_ mass and species in Kuwait. The study aims to compare the indoor-to-outdoor ratios of mass and elements during dust events and non-dust events and to investigate the ratios of coarse-to-fine particle mass concentrations and elements indoors and outdoors. 

## 2. Materials and Methods

### 2.1. Home Selection

**Figure 1:**
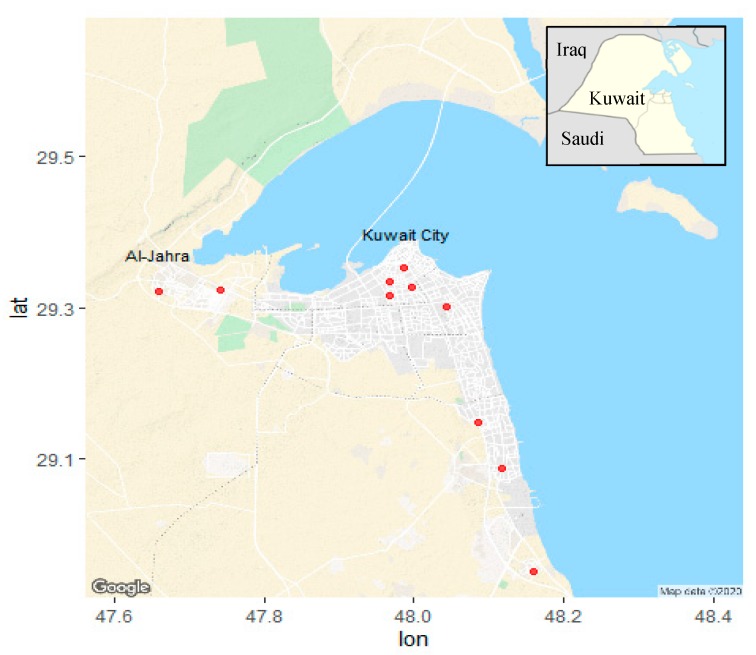
Geographical distribution of sampled residencies in Kuwait as illustrated by red dots, plotted using *ggmap* [27].

Ten typical residences located in the Kuwait City metropolitan area were studied during the period between September 2017 and March 2018. Weekly samples were collected at different times during the autumn and winter season. Some of the homes were sampled twice. Locations of the houses are illustrated by dark red squares in Figure 1. Homes were selected based on the willingness of owners to participate in the study and the absence of major indoor particle sources (e.g., smokers, traditional incense burners) and air purifiers. Our original study design included 30 homes; however, funding was not available after the first year of the study. Due to the limited number of samples, our study focused on houses occupied only by Kuwaiti nationals. In one location site, the height of the sampling head was 0.3 m shorter than an adjacent wall (Figure S2). To ensure that this deviation from the sampling protocol did not affect our results, we ran a separate analysis excluding data from that home. 

### 2.2. Sampling Techniques

Indoor and outdoor PM_10_ and PM_2.5_ samples were collected on Teflon membrane filters (Pall Laboratory, diameter: 37 mm, pore size: 2 µm) using a custom-made Harvard sampler [28] at a sampling flow rate of 5 L/min for 7 days (Figures S1 and S2). This sampler uses size-selective inlets with polyurethane foam (PUF) substrates to remove particles with sizes above certain values (i.e., cut-points). For each cut-point, the sampler had two impaction stages (with the same cut-point) in series to remove particles above the cut-point, allowing for the collection of high-loading particles during dust storm events under extreme temperature conditions. Based on previously published tests, the PUF impaction substrates employed in this study have an estimated capacity of about 10 mg of dust per impaction stage, which enables the outdoor samplers to function accurately in challenging desert environments [29]. These samplers were particularly suitable for use in Kuwait City, which is located in an arid area experiencing frequent dust storms and high temperatures [28]. 

Each sampler was placed inside and outside the study homes by a field technician one to two days prior to the beginning of the sampling. For each house, the indoor samplers were positioned in a major activity room of the residents (e.g., the living room). The outdoor samplers were placed outside of the house at a location at least three meters away from the exterior wall (Figures S1 and S2). Samplers automatically start collection when they are plugged into power and they stopped when we unplugged them at the end of each sampling week. For each house, the indoor and outdoor sampling were initiated together at the same time. We stopped all samplers at the same time of the day as the initiation time for each house (usually between 9 and 9:30 am in the morning). The particles collected on the filters were analyzed for: (1) PM_10_ and PM_2.5_ mass by gravimetric analysis (MT-5, Mettler Toledo, Columbus); (2) trace elements by energy-dispersive X-ray fluorescence spectrometry (XRF; Epsilon 5, PANalytical, the Netherland); and (3) black carbon (BC) by optical transmission measurement (OT21, Magee Scientific, Berkeley, CA, USA). All analyses were conducted at the Harvard T.H. Chan School of Public Health. 

### 2.3. Dust Event Identification

Because Fe is one of the major crustal elements, it was used as a tracer of dust storm events. Under heavy filter loadings, XRF for Fe performs better than other crustal elements. Since the median Fe concentration in outdoor PM_2.5_ samples was 0.8 μg/m^3^, this level was used as a threshold for dust events (Figure S5). That is, outdoor PM_2.5_ weekly samples with a Fe concentration ≥ 0.8 μg/m^3^ were categorized as samples with “dust event present”. Whereas, outdoor PM_2.5_ weekly samples with a Fe concentration < 0.8 μg/m^3^ were categorized as samples with “dust event absent”. 

### 2.4. Home Tightness Analysis

The following equation quantifies the indoor to outdoor ratio of a fine (f) element X at home j, [I/O]_xfj_:

[I/O]_xfj_ = X_fij_/X_foj_, (1)

where X_fij_ represents the indoor PM_2.5_ concentration of a trace element X measured at home j and X_foj_ represents the outdoor fine particle concentration of element X measured outside home j during the same period. We matched each indoor and outdoor sample to the home and used the ratios calculated for each home as a proxy for home tightness. The indoor-to-outdoor (I/O) ratios for PM_10_ during dust and non-dust storm events were not compared because of the limited number of valid outdoor PM_10_ samples. Instead, the ratios of coarse-to-fine elemental concentrations both indoors and outdoors were estimated as an alternative approach, allowing us to compare the behavior of coarse and fine particles. 

### 2.5. Coarse-to-Fine Particle Ratio Analysis 

This analysis first calculated indoor (i) coarse-to-fine particle ratios for an element X and home j, [C/F]_xij_, as follows:

[C/F]_xij_ = X_cij_/X_fij_, (2)

where coarse (c) concentration is defined as PM_10_ concentration minus PM_2.5_ concentration. X_cij_ represents the indoor (i) coarse (c) concentration of an element X for home j and X_fij_ represents the indoor fine (f) concentration of element X for home j. Coarse elemental concentrations, X_cij_, were determined by subtracting the elemental concentrations of indoor PM_2.5_ from the corresponding ones of indoor PM_10_. 

The outdoor coarse-to-fine particle ratios for an element X and home j, [C/F]_xoj_, are estimated as shown below:

[C/F]_xoj_ = X_coj_/X_foj_, (3)

where X_coj_ represents the outdoor coarse concentration of an element X for home j and X_foj_ represents the outdoor fine concentration of element X for home j. Outdoor coarse elemental concentrations, X_cio_, are determined by subtracting the elemental concentrations of outdoor PM_2.5_ from the corresponding ones of outdoor PM_10_. To examine differences between fine and coarse particles, we selected six terrestrial elements of which their concentrations are known to be considerably higher during dust storm events: Fe, Ca, Si, Al, Ti, and K.

### 2.6. Statistical Analysis

We used “week” as the temporal unit of analysis. The distributions of indoor and outdoor concentrations were characterized by means, standard deviations, medians, and interquartile ranges. The normality of data was examined using a Shapiro-Wilks test and graphical explorations. The difference between indoor fine particles during dust event weeks and non-dust event weeks and the difference between indoor and outdoor C/F ratios of elements were tested. To make inferential statistical tests, t-tests were employed to compare the difference in means. The analyses were repeated on log-transformed means and using a non-parametric Wilcoxon test of medians. All tests were two-sided with pre-specified alpha at the 0.05 level. All analyses were done using R version 3.5.2.

## 3. Results

Indoor and outdoor particulate matter samples were collected at 10 residential homes located in Kuwait City during the period between September 2017 and March 2018. Two samples with pump failure were excluded from the analysis. Of the 10 houses, 3 were sampled twice, sequentially after we found that air purifiers were used. We removed the samples with air purifiers. In total, the data analysis included 13 indoor PM_2.5_ samples, 13 outdoor PM_2.5_ samples, 7 indoor PM_10_ samples, and 7 outdoor PM_10_ samples.

This analysis included particle mass, BC, and 19 trace elements (Na, Mg, Al, Si, S, Cl, K, Ca, Ti, V, Cr, Mn, Fe, Ni, Cu, Zn, Br, Sr, and Zr). Table 1 presents the distribution statistics of the indoor and outdoor PM_2.5_ levels of mass and species and their mean I/O concentration ratios (Table S1 in the supplemental material presents the corresponding medians and distributions). In general, indoor concentrations were lower than those outdoors. Inside homes fine concentrations of S (2.556 µg/m^3^) and BC (2.134 µg/m^3^) were the highest compared to the other species. Outside homes fine concentrations of S (4.450 µg/m^3^), BC (3.672 µg/m^3^), and Ca (1.452 µg/m^3^) were the highest. Both indoor and outdoor mean concentrations of all other elements were below 1 µg/m^3^. Finally, BC and elemental I/O ratios ranged from 0.31 to 0.64, except for Cl, which was 1.10.

Indoor and outdoor concentrations of PM_10_ mass and species, as well as their I/O concentration ratios, are summarized in Table 2 (and Table S2). Similar to PM_2.5_, indoor mean mass and elemental concentrations of PM_10_ mass and species were lower than those outdoors. S, BC, Si, and Ca were the most abundant, ranging from 1.888 to 2.781 µg/m^3^, while all other elements were below 1 µg/m^3^. For outdoor PM_10_ samples, Ca, Si, and BC were the most abundant, ranging from 4.668 to 8.302 µg/m^3^, while Cr, Ni, and Zr had the lower concentrations. The highest I/O concentration ratios were observed for Na and S, which were 0.89 and 0.78, respectively. The lowest I/O concentration ratio of 0.22 was observed for Ca and Cr. 

**Figure 2:**
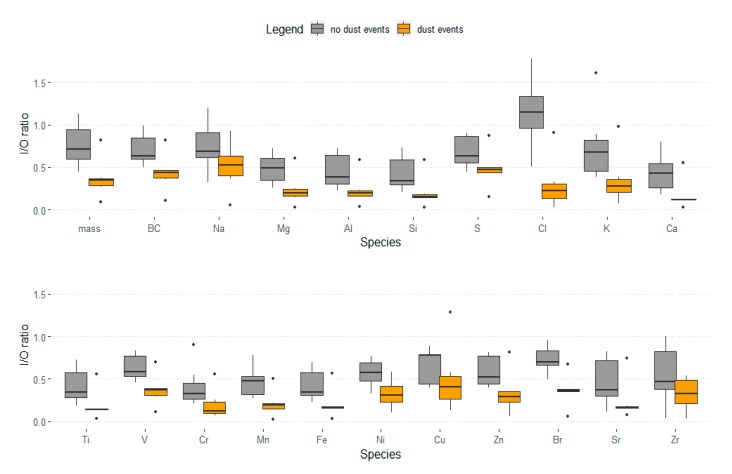
Box plots illustrate the difference between I/O concentration ratios of PM_2.5_ mass, BC, and 19 elements during dust and non-dust events.

Figure 2 illustrates the results of *t*-tests comparing I/O concentration ratios for PM_2.5_ mass and species during dust and non-dust events (Table S3 shows results from all statistical tests). The weekly dust event ratios of mass, BC, Mg, Cl, Ca, V, Mn, Fe, Ni, and Br were significantly lower than those during non-dust event weeks (*p* < 0.05). Other elements showed lower PM_2.5_ I/O ratios during dust storm weeks as compared to those during non-dust storm weeks. However, the differences were not statistically significant [Na (*p* = 0.167), Al (*p* = 0.060), Si (*p* = 0.063), S (*p* = 0.118), K (*p* = 0.091), Ti (*p* = 0.056), Cr (*p* = 0.110), Cu (*p* = 0.466), Zn (*p* = 0.071), Sr (*p* = 0.140), and Zr (*p* = 0.159)]. In general, I/O ratios were lower during dust storm weeks (0.2–0.3), as compared to those during non-dust storm events (0.4–0.6). Remarkably, fine Cl represented a considerably higher I/O ratio (1.10) during normal weather. Finally, *t*-test analysis was performed to compare indoor PM_2.5_ mass concentrations during weeks with and without dust events and suggested a large difference: 18.0 µg/m^3^ during dust event weeks and 37.5 µg/m^3^ during non-dust event weeks (*p* = 0.04).

**Figure 3:**
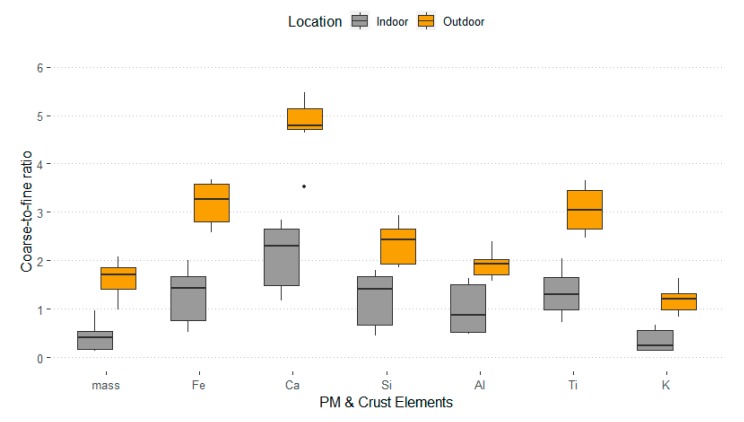
Coarse-to-fine particle ratios of mass and elements measured indoors and outdoors.

Table 3 shows the mean concentrations of mass, Fe, Ca, Si, Al, Ti, and K in the fine and coarse particle modes measured indoors and outdoors. For coarse trace element particles, Ca had the highest indoor and outdoor mean concentrations, 1.346 and 6.829 µg/m^3^, respectively, while Ti had the lowest indoor and outdoor concentrations, 0.036 and 0.171 µg/m^3^, respectively. For fine trace element particles, Si had the highest indoor and outdoor mean concentrations, 0.865 and 2.142 µg/m^3^, respectively, while similarly, Ti had the lowest in indoor and outdoor concentrations, 0.023 and 0.058 µg/m^3^, respectively. The indoor coarse-to-fine elemental ratios were generally lower than the corresponding outdoor ratios. Ca had the highest coarse-to-fine ratio both indoors and outdoors, 2.1 and 4.8, respectively. Finally, K exhibited the lowest coarse-to-fine ratios among all six elements because it is not exclusively a crustal element like the other five elements are. Two-tailed t-tests found significant differences between coarse-to-fine elemental ratios indoor and outdoor for particle mass (*p* < 0.001), Fe (*p* < 0.001), Ca (*p* < 0.001), Si (*p* = 0.001), Al (*p* = 0.003), Ti (*p* < 0.001), and K (*p* < 0.001) (Table S4). For mass and all these terrestrial elements, indoor ratios were clearly higher than indoor coarse-to-fine ratios (Figure 3).

Excluding data from the location site where there was a deviation from the sampling protocol did not change our findings (Figures S3 and S4).

## 4. Discussion

This indoor/outdoor study in Kuwait City examined the relationship between home insulation and PM_2.5_ I/O concentration ratios of mass for BC and 19 elements. Mass and all of these 20 species exhibited lower PM_2.5_ I/O ratios during dust event weeks, most within the 20% to 30% range, as compared to non-dust event weeks, which mostly fell within the 40% to 60% range. The PM_2.5_ I/O ratios of mass, BC, Mg, Cl, Ca, V, Mn, Fe, Ni, and Br concentrations during dust event weeks were significantly lower than the corresponding ratios during non-dust storm weeks (Figure 2). In addition, indoor PM_2.5_ mass concentrations were even lower during dust storm weeks compared to levels during non-dust storm weeks. These results suggest that home tightening practices implemented by residents during dust events have a protective effect against particle penetration. These practices include keeping windows and doors tightly closed during severe dust storms and residents also taping the casings of their windows and placing wet towels under their doors. 

Our results are consistent with several previous studies, which estimated associations between decreased natural ventilation and reduced PM_2.5_ mass concentrations indoors in residential and commercial buildings [30,31]. Another PM_2.5_ elemental concentration study conducted in residential homes in Finland similarly found higher elemental levels during warmer seasons when residents open their windows and lower elemental levels during cold seasons when residents close their windows [32]. Similarly, a study in Qatar found a difference in indoor PM concentrations in an office building during the days that the building was open to the public and employees [26].

Chlorine and copper can have indoor sources affecting their I/O ratio. We observed a considerably higher PM_2.5_ I/O ratio for Cl (1.10). A likely explanation is the resuspension of dust and sea salt particles originated from outdoors and settling on indoor surfaces, which was observed in a previous study in Finland [32]. However, indoor Cl could also be associated with bleach-containing products used for sanitizing and/or cleaning [33]. Another study in China reported a low Cl I/O ratio of 0.5 at homes in Beijing, but this could be attributed to the terrestrial location of Beijing [34]. The PM_2.5_ indoor-to-outdoor ratio of Cu comparing dust storm events to non-dust storm events showed a considerably higher *p*-value than 0.05. This may be because Cu has important indoor sources such as electric motor brushes used for refrigerators or vacuum cleaners [35,36,37]. 

Coarse-to-fine ratios were compared to investigate differences in the penetration ratios between indoors and outdoors. The study observed statistically significant differences in mass and the concentrations of crustal elements (Fe, Ca, Si, Al, Ti, and K) between indoors and outdoors. These crustal elements are abundant in the air during dust storms, so understanding their indoor behavior is essential for dust exposure prevention. Coarse particles displayed overall higher outdoor concentrations compared to those indoors for both mass and elemental concentrations (Table 3). The outdoor coarse-to-fine particle ratios for all six terrestrial elements were higher compared to their corresponding indoor ratios (Figure 3). These results suggest that penetration efficiencies of coarse particles of crustal elements are lower than those of fine particles of the same elements. Notably, this finding is in agreement with those reported by previous studies and is because of the larger size of coarse particles [33,34,35,36]. Due to their larger size, coarse particles have a lower indoor concentration as they have a lower ability to penetrate residencies through window and door cracks. Also, due to their higher settling velocities, coarse particles deposit faster (higher impaction rate) on indoor surfaces as compared to fine particles, which contribute to their lower indoor levels [38,39,40,41]. 

This study successfully measured PM_2.5–10_ and PM_2.5_ mass and elemental concentrations both inside and outside residential houses. The custom-designed Harvard samplers enabled us to conduct accurate particle measurements under extreme conditions of weather and heavy particle loadings during dust storm episodes. Further, because a more extended sampling period was used for this study, many elements were above the respective detection limits and were included in the analysis. Each weekly sample was assigned as either being a dust storm week or a non-dust storm week. The tradeoff of using weekly samples is the potential underestimation of dust storm effects. For example, some dust storm weeks might have had five days of dust, while others could have had just one day. Due to our limited sample size, we were not able to control for the number of days of dust storms within any given week. 

Using Fe to separate dust events from non-dust events may not be optimal in the presence of real-time dust data. Further validation of the median Fe threshold is warranted. Furthermore, we used the I/O ratios for each home as a proxy to home tightness. Using this proxy to make inferences about home tightness can be justified since we are comparing each home to itself. However, conclusions must be treated with caution since we did not control for between-house variables due to the small sample size. Unfortunately, due to the unexpected cut in funding after the first year of our study, we failed to maintain a larger sample size. This may have limited the power and generalizability of the study. However, the results were consistent, and the effect size was large enough to detect a significant difference. A further investigation of particle physical outdoor/indoor behaviors of every trace element is warranted. We did not collect logs of the entrances and exits of individuals in each home. This prevented us from understanding whether the low I/O during dust storms can be adequately explained by individuals staying indoors and not leaving.

In conclusion, our study is the first to simultaneously measure indoor and outdoor PM_10_ and PM_2.5_ during dust and non-dust storm weeks in Kuwait. This investigation provided more granularity in quantifying the indoor-to-outdoor ratios for particulate mass and species with population-based scope, large spatial coverage, and high-quality custom-designed samplers in Kuwait. Since houses were sealed tightly during dust storms, I/O ratios of species were lower during dust storm weeks than during non-dust storm weeks. This study also found that the indoor ratios of coarse-to-fine particle concentrations were different from those outdoor, which can be attributed to restricted penetration of coarse particles due to their larger sizes and more rapid gravitational deposition as compared to fine. Findings from this study could aid local authorities in initiating and facilitating public health policy development and prevention of natural source pollutants such as warning systems and advisories to stay indoors. However, this study was based only on data collected from Kuwait, a developed country with well-constructed homes that can be kept tightly closed. Further studies on crustal particles from dust storms and their penetration should be conducted in less developed countries in the Middle East where homes may be leaky and without central ventilation systems. 

## Figures and Tables

**Table 1:** Summary of mean and standard deviation indoor and outdoor PM_2.5_ mass and species concentrations and their indoor-to-outdoor (I/O) ratios.

Species	Indoor Concentration N = 13		Outdoor Concentration N = 13		I/O Ratio
	Mean	SD	Mean	SD	
**Mass**	28.52	17.94	44.34	8.789	0.70
**BC**	2.134	0.930	3.672	0.865	0.59
**Na**	0.234	0.096	0.388	0.112	0.64
**Mg**	0.113	0.069	0.328	0.085	0.37
**Al**	0.320	0.231	0.950	0.339	0.36
**Si**	0.644	0.531	2.072	0.787	0.33
**S**	2.556	1.212	4.450	1.866	0.59
**Cl**	0.035	0.032	0.050	0.046	1.10
**K**	0.239	0.182	0.436	0.109	0.57
**Ca**	0.421	0.358	1.452	0.484	0.31
**Ti**	0.018	0.014	0.059	0.020	0.32
**V**	0.008	0.003	0.016	0.004	0.52
**Cr**	0.001	0.001	0.003	0.001	0.32
**Mn**	0.006	0.004	0.020	0.005	0.35
**Fe**	0.252	0.176	0.816	0.249	0.33
**Ni**	0.003	0.001	0.006	0.002	0.46
**Cu**	0.011	0.008	0.019	0.008	0.58
**Zn**	0.039	0.020	0.091	0.060	0.48
**Br**	0.013	0.006	0.023	0.006	0.56
**Sr**	0.003	0.002	0.009	0.002	0.37
**Zr**	0.002	0.001	0.005	0.001	0.45

**Table 2:** Summary of means and standard deviations indoor and outdoor PM_10_ mass and species concentrations and their I/O ratios.

Species	Indoor Concentration N = 7		Outdoor Concentration N = 7		I/O Ratio
	Mean	SD	Mean	SD	
**Mass**	40.55	22.99	116.8	18.38	0.34
**BC**	2.668	0.974	4.668	1.173	0.58
**Na**	0.293	0.076	0.345	0.095	0.89
**Mg**	0.295	0.213	0.988	0.153	0.30
**Al**	0.917	0.768	2.710	0.646	0.32
**Si**	2.082	1.870	6.936	1.673	0.29
**S**	2.781	0.734	3.813	1.461	0.78
**Cl**	0.061	0.033	0.197	0.194	0.44
**K**	0.444	0.313	1.030	0.209	0.43
**Ca**	1.888	1.790	8.302	1.509	0.22
**Ti**	0.059	0.054	0.228	0.048	0.25
**V**	0.011	0.004	0.023	0.003	0.48
**Cr**	0.003	0.003	0.014	0.002	0.22
**Mn**	0.017	0.013	0.066	0.014	0.27
**Fe**	0.783	0.681	3.264	0.641	0.23
**Ni**	0.004	0.002	0.015	0.002	0.31
**Cu**	0.019	0.013	0.042	0.020	0.48
**Zn**	0.061	0.017	0.173	0.107	0.45
**Br**	0.018	0.005	0.031	0.006	0.60
**Sr**	0.011	0.009	0.047	0.009	0.24
**Zr**	0.004	0.005	0.015	0.006	0.27

**Table 3:** Mean indoor and outdoor concentrations and I/O ratios for coarse and fine particle mass, Fe, Ca, Si, Al, Ti, and K measured in 7 matched indoor/outdoor paired samples in Kuwait.

Species	Coarse Indoor (μg/m^3^)	Coarse Outdoor (μg/m^3^)	Fine Indoor (μg/m^3^)	Fine Outdoor (μg/m^3^)	Indoor Ratio ^a^	Outdoor Ratio ^b^
**Mass**	13.43	71.74	27.12	45.04	0.4	1.6
**Fe**	0.474	2.471	0.309	0.792	1.3	3.2
**Ca**	1.346	6.829	0.542	1.473	2.1	4.8
**Si**	1.218	4.794	0.865	2.142	1.2	2.3
**Al**	0.499	1.759	0.418	0.951	1.0	1.9
**Ti**	0.036	0.171	0.023	0.058	1.3	3.1
**K**	0.124	0.551	0.320	0.479	0.4	1.2

^a^ Ratio of coarse to fine concentrations indoors (dimensionless). ^b^ Ratio of coarse to fine concentrations outdoors (dimensionless).

## Supplementary Materials

The following are available online at https://www.mdpi.com/1660-4601/17/7/2433/s1, Table S1: Summary of median and distribution of indoor and outdoor PM_2.5_ species concentrations. Table S2: Summary of median and distribution of indoor and outdoor PM_10_ species concentrations. Figure S1: Harvard samplers collecting PM_10_ and PM_2.5_ inside a Kuwait home. Figure S2: Harvard samplers collecting PM_10_ and PM_2.5_ outside a Kuwait home. Table S3: *p*-values from Shapiro-Wilks test of normality, t-test with identity means, t-test with log-transformed means, and Wilcoxon test, comparing fine element indoor to outdoor concentration in dust events versus no dust events (Corresponds to Figure 2). Table S4: *p*-values from Shapiro-Wilks test of normality, t-test with identity means, t-test with log-transformed means, and Wilcoxon test, comparing coarse-to-fine mean ratios in indoor versus outdoor. (Corresponds to Figure 3). Figure S3: Box plots illustrate the difference between I/O concentration ratios of PM_2.5_ mass, BC and 19 elements during dust and non-dust events after excluding H2. Figure S4: Box plot illustrates coarse-to-fine particle ratios of mass and elements measured indoors and outdoors after excluding H2. Figure S5: Histogram illustrates the median of outdoor fine Fe concentrations that we utilized as a threshold for dust storm events identification.
