# Supplementary material for: Dust Events and Indoor Air Quality in Residential Homes in Kuwait
Source: Int J Environ Res Public Health. 2020 Apr 3;17(7):2433. doi: 10.3390/ijerph17072433 (PMC7178282; doi:10.3390/ijerph17072433)
Supplement: Supplementary file 1 [file ijerph-17-02433-s001.pdf]

## Supplemental Material:

**Table S1:** Summary of median and distribution of indoor and outdoor $PM_{2.5}$ species concentrations

| Species | Indoor Concentration N=13 | | | | Outdoor Concentration N=13 | | | |
|---|---|---|---|---|---|---|---|---|
| | Median | IQR | Min | Max | Median | IQR | Min | Max |
| **Mass** | 23.85 | 20.67 | 3.901 | 74.36 | 43.78 | 9.397 | 28.74 | 58.07 |
| **BC** | 2.073 | 0.961 | 0.378 | 4.192 | 3.379 | 0.961 | 2.795 | 5.691 |
| **Na** | 0.232 | 0.087 | 0.020 | 0.419 | 0.413 | 0.119 | 0.245 | 0.609 |
| **Mg** | 0.097 | 0.080 | 0.014 | 0.275 | 0.327 | 0.130 | 0.195 | 0.457 |
| **Al** | 0.236 | 0.160 | 0.052 | 0.919 | 0.830 | 0.459 | 0.560 | 1.559 |
| **Si** | 0.510 | 0.413 | 0.080 | 2.138 | 1.734 | 1.096 | 1.125 | 3.611 |
| **S** | 2.406 | 1.461 | 0.526 | 5.199 | 3.727 | 1.900 | 2.333 | 8.639 |
| **Cl** | 0.036 | 0.031 | 0.002 | 0.117 | 0.041 | 0.029 | 0.010 | 0.187 |
| **K** | 0.185 | 0.113 | 0.035 | 0.666 | 0.420 | 0.128 | 0.279 | 0.687 |
| **Ca** | 0.252 | 0.361 | 0.048 | 1.336 | 1.365 | 0.508 | 0.814 | 2.431 |
| **Ti** | 0.012 | 0.014 | 0.002 | 0.052 | 0.055 | 0.028 | 0.035 | 0.094 |
| **V** | 0.009 | 0.004 | 0.002 | 0.013 | 0.016 | 0.005 | 0.012 | 0.027 |
| **Cr** | 0.001 | 0.000 | 0.000 | 0.002 | 0.003 | 0.001 | 0.002 | 0.004 |
| **Mn** | 0.006 | 0.004 | 0.001 | 0.013 | 0.019 | 0.008 | 0.012 | 0.030 |
| **Fe** | 0.186 | 0.160 | 0.033 | 0.663 | 0.779 | 0.367 | 0.542 | 1.272 |
| **Ni** | 0.003 | 0.002 | 0.001 | 0.004 | 0.006 | 0.002 | 0.004 | 0.009 |
| **Cu** | 0.008 | 0.005 | 0.002 | 0.031 | 0.018 | 0.010 | 0.010 | 0.037 |
| **Zn** | 0.045 | 0.033 | 0.003 | 0.067 | 0.067 | 0.038 | 0.034 | 0.251 |
| **Br** | 0.014 | 0.008 | 0.001 | 0.023 | 0.024 | 0.005 | 0.014 | 0.036 |
| **Sr** | 0.002 | 0.002 | 0.001 | 0.008 | 0.009 | 0.003 | 0.005 | 0.012 |
| **Zr** | 0.002 | 0.001 | 0.000 | 0.004 | 0.004 | 0.002 | 0.003 | 0.007 |

Concentrations are expressed in $\mu g/m^3$

**Table S2:** Summary of median and distribution of indoor and outdoor $PM_{10}$ species concentrations

| Species | Indoor Concentration N=7 | | | | Outdoor Concentration N=7 | | | |
|---|---|---|---|---|---|---|---|---|
| | Median | IQR | Min | Max | Median | IQR | Min | Max |
| **Mass** | 35.11 | 28.06 | 18.31 | 76.46 | 119.5 | 16.75 | 86.76 | 145.6 |
| **BC** | 2.683 | 1.185 | 1.521 | 4.293 | 4.139 | 1.420 | 3.728 | 6.683 |
| **Na** | 0.284 | 0.071 | 0.208 | 0.430 | 0.322 | 0.124 | 0.193 | 0.462 |
| **Mg** | 0.273 | 0.273 | 0.089 | 0.669 | 1.042 | 0.227 | 0.800 | 1.203 |
| **Al** | 0.672 | 0.791 | 0.207 | 2.416 | 2.524 | 0.527 | 2.144 | 4.018 |
| **Si** | 1.568 | 1.747 | 0.418 | 5.799 | 6.478 | 1.408 | 5.481 | 10.331 |
| **S** | 2.560 | 1.155 | 1.833 | 3.669 | 4.173 | 1.600 | 2.140 | 6.400 |
| **Cl** | 0.072 | 0.035 | 0.014 | 0.102 | 0.144 | 0.022 | 0.029 | 0.626 |
| **K** | 0.289 | 0.390 | 0.208 | 0.949 | 0.926 | 0.174 | 0.853 | 1.393 |
| **Ca** | 1.390 | 2.148 | 0.343 | 5.121 | 7.827 | 1.585 | 7.082 | 10.999 |
| **Ti** | 0.043 | 0.066 | 0.015 | 0.158 | 0.212 | 0.046 | 0.189 | 0.324 |
| **V** | 0.011 | 0.005 | 0.007 | 0.017 | 0.024 | 0.004 | 0.018 | 0.026 |
| **Cr** | 0.002 | 0.003 | 0.001 | 0.007 | 0.014 | 0.002 | 0.010 | 0.017 |
| **Mn** | 0.012 | 0.018 | 0.006 | 0.038 | 0.060 | 0.018 | 0.050 | 0.086 |
| **Fe** | 0.596 | 0.841 | 0.231 | 1.995 | 3.064 | 0.644 | 2.559 | 4.331 |
| **Ni** | 0.004 | 0.003 | 0.003 | 0.008 | 0.015 | 0.002 | 0.010 | 0.017 |
| **Cu** | 0.012 | 0.015 | 0.008 | 0.041 | 0.035 | 0.027 | 0.023 | 0.072 |
| **Zn** | 0.059 | 0.026 | 0.041 | 0.087 | 0.129 | 0.105 | 0.080 | 0.377 |
| **Br** | 0.020 | 0.007 | 0.011 | 0.024 | 0.029 | 0.003 | 0.026 | 0.044 |
| **Sr** | 0.010 | 0.013 | 0.003 | 0.025 | 0.046 | 0.010 | 0.032 | 0.061 |
| **Zr** | 0.004 | 0.007 | 0.000 | 0.011 | 0.012 | 0.010 | 0.010 | 0.022 |

Concentrations are expressed in $\mu g/m^3$

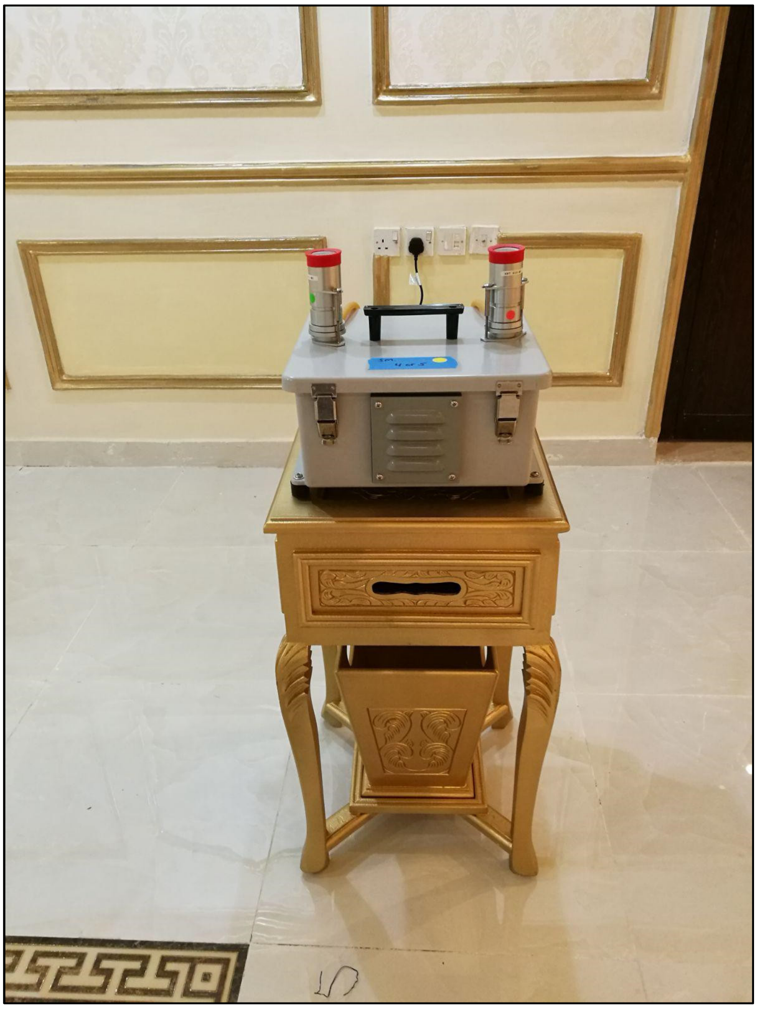

**Figure S1:** Harvard samplers collecting $PM_{10}$ and $PM_{2.5}$ inside a Kuwaiti home.

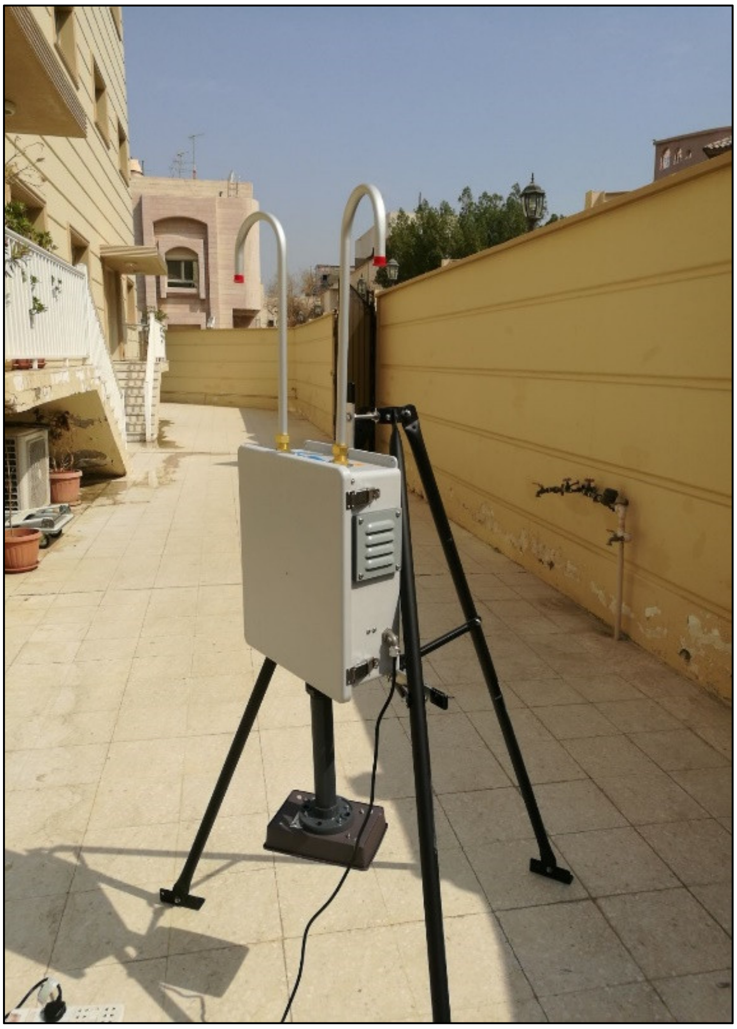

**Figure S2:** Harvard samplers collecting $PM_{10}$ and $PM_{2.5}$ outside a Kuwaiti home.

**Table S3:** P-values from Shapiro-Wilks test of normality, t-test with identity means, t-test with log-transformed means, and Wilcoxon test, comparing fine element indoor to outdoor concentration in dust events versus no dust events (Corresponds to Figure 2)

| Species | Shapiro-Wilks test | t-test | log t-test | Wilcoxon[a] |
|---|---|---|---|---|
| **Mass** | 0.012 | 0.042 | 0.019 | 0.014 |
| **BC** | 0.943 | 0.040 | 0.075 | 0.022 |
| **Na** | 0.999 | 0.167 | 0.228 | 0.138 |
| **Mg** | 0.548 | 0.038 | 0.059 | 0.022 |
| **Al** | 0.169 | 0.060 | 0.063 | 0.022 |
| **Si** | 0.200 | 0.063 | 0.054 | 0.022 |
| **S** | 0.146 | 0.118 | 0.135 | 0.101 |
| **Cl** | 0.104 | 0.005 | 0.005 | 0.002 |
| **K** | 0.100 | 0.091 | 0.055 | 0.035 |
| **Ca** | 0.160 | 0.049 | 0.035 | 0.022 |
| **Ti** | 0.102 | 0.056 | 0.047 | 0.022 |
| **V** | 0.868 | 0.021 | 0.046 | 0.014 |
| **Cr** | 0.049 | 0.110 | 0.047 | 0.073 |
| **Mn** | 0.691 | 0.021 | 0.050 | 0.014 |
| **Fe** | 0.262 | 0.049 | 0.053 | 0.022 |
| **Ni** | 0.864 | 0.024 | 0.046 | 0.051 |
| **Cu** | 0.361 | 0.466 | 0.231 | 0.234 |
| **Zn** | 0.266 | 0.071 | 0.077 | 0.035 |
| **Br** | 0.728 | 0.004 | 0.047 | 0.005 |
| **Sr** | 0.018 | 0.140 | 0.093 | 0.101 |
| **Zr** | 0.411 | 0.159 | 0.452 | 0.366 |

[a] We were not able to rely on Wilcoxon tests due to the high number of ties

**Table S4:** P-values from Shapiro-Wilks test of normality, t-test with identity means, t-test with log-transformed means, and Wilcoxon test, comparing coarse-to-fine mean ratios in indoor versus outdoor. (Corresponds to Figure 3)

| Elements | Shapiro Wilks | t-test | log t-test | Wilcoxon[a] |
|---|---|---|---|---|
| **Mass** | 0.157 | <0.001 | 0.001 | <0.001 |
| **Fe** | 0.250 | <0.001 | 0.002 | 0.001 |
| **Ca** | 0.114 | <0.001 | <0.001 | 0.001 |
| **Si** | 0.425 | 0.001 | 0.011 | 0.001 |
| **Al** | 0.107 | 0.003 | 0.011 | 0.007 |
| **Ti** | 0.405 | <0.001 | 0.000 | 0.001 |
| **K** | 0.451 | <0.001 | 0.002 | 0.001 |

[a] We were not able to rely on Wilcoxon tests due to the high number of ties

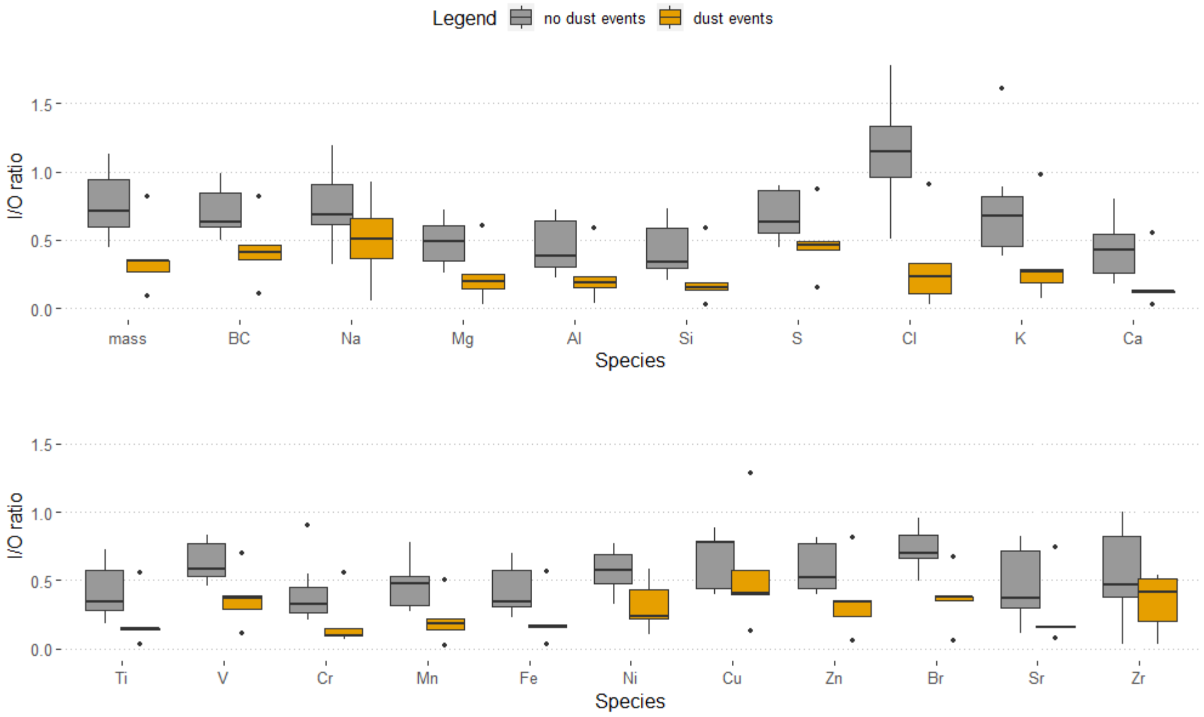


**Figure S3:** Box plots illustrate the difference between I/O concentration ratios of $PM_{2.5}$ mass, BC and 19 elements during dust and non-dust events after excluding H2.

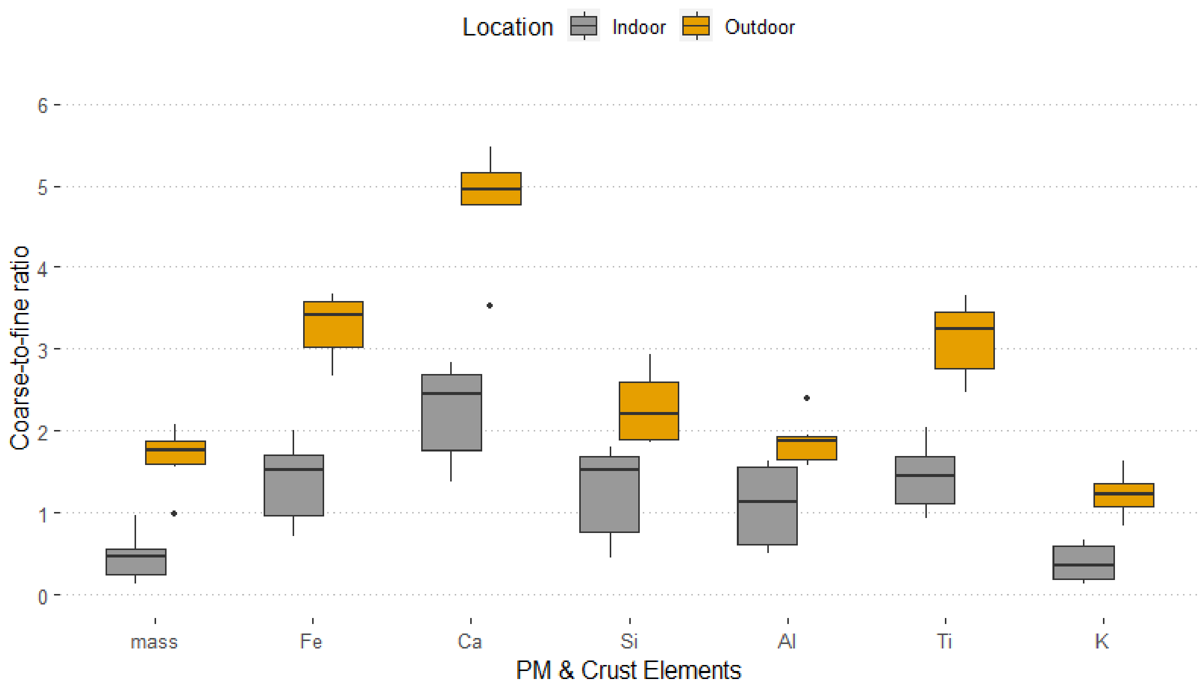


**Figure S4:** Box plot illustrates coarse-to-fine particle ratios of mass and elements measured indoors and outdoors after excluding H2.

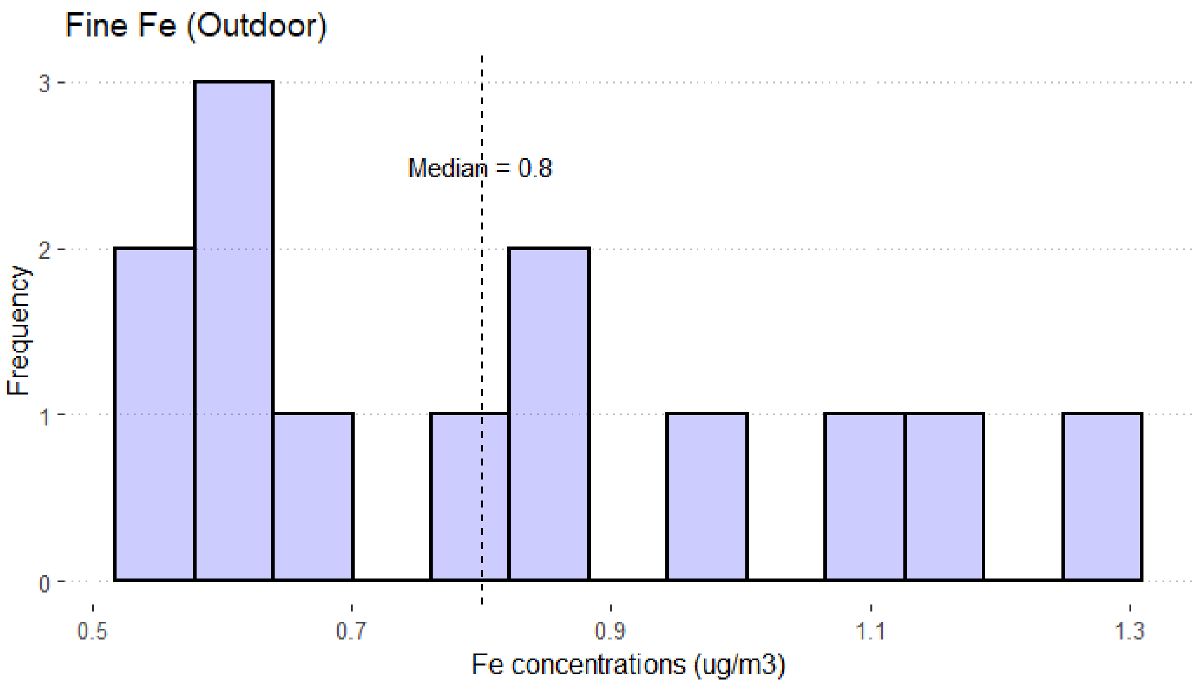


**Figure S5:** Histogram illustrates the median of outdoor fine Fe concentrations that we utilized as a threshold for dust storm events identification.
